# Identification of structural determinants on tau protein essential for its pathological function: novel therapeutic target for tau immunotherapy in Alzheimer’s disease

**DOI:** 10.1186/alzrt277

**Published:** 2014-08-01

**Authors:** Eva Kontsekova, Norbert Zilka, Branislav Kovacech, Rostislav Skrabana, Michal Novak

**Affiliations:** 1 Axon Neuroscience SE, Dvorakovo nabrezie 10, 811 02 Bratislava, Slovak Republic; 2 Present address: Institute of Neuroimmunology, Dubravska cesta 9, 84510 Bratislava, Slovak Republic

## Abstract

**Introduction:**

Pathologically modified tau protein is the main feature of Alzheimer’s disease (AD) and related tauopathies. Therefore, immunotherapies that target mis-disordered tau represent a promising avenue for the disease-modifying treatment of AD. In this report, we present our discovery of (1) a novel target for tau immunotherapy; (2) monoclonal antibody DC8E8, which neutralizes this target; and (3) the results of efficacy studies of DC8E8 in a murine model of tauopathy.

**Methods:**

*In vitro* tau oligomerisation assays were used for the selection of antibodies. The therapeutic efficacy of DC8E8 was evaluated in transgenic mice. The structure of the DC8E8 epitope was determined by X-ray crystallography.

**Results:**

Screening of a panel of monoclonal antibodies for their inhibitory activity in an *in vitro* pathological tau–tau interaction assay yielded DC8E8, which reduced the amount of oligomeric tau by 84%. DC8E8 recognised all developmental stages of tau pathology in AD human brains, including pretangles and intra- and extracellular tangles. Treatment with DC8E8 in a mouse AD model expressing mis-disordered human tau significantly reduced the amount of insoluble oligomerised tau and the number of early and mature neurofibrillary tangles in the transgenic mouse brains. By using a panel of tau-derived peptides in a competitive enzyme-linked immunosorbent assay, we identified the tau domain essential for pathological tau–tau interaction, which is targeted by DC8E8. The antibody was capable of binding to four highly homologous and yet independent binding regions on tau, each of which is a separate epitope. The X-ray structure of the DC8E8 Fab apo form, solved at 3.0 Å, suggested that the four DC8E8 epitopes form protruding structures on the tau molecule. Finally, by kinetic measurements with surface plasmon resonance, we determined that antibody DC8E8 is highly discriminatory between pathological and physiological tau.

**Conclusions:**

We have discovered defined determinants on mis-disordered truncated tau protein which are responsible for tau oligomerisation leading to neurofibrillary degeneration. Antibody DC8E8 reactive with these determinants is able to inhibit tau–tau interaction *in vitro* and *in vivo*. DC8E8 is able to discriminate between the healthy and diseased tau proteome, making its epitopes suitable targets, and DC8E8 a suitable candidate molecule, for AD immunotherapy.

## Introduction

Neurodegenerative foldopathies represent a group of human protein-misfolding disorders that are characterised by a pathological alteration in conformation of a native protein which makes it resistant to degradation and leads to pathological gain and loss of function. These are followed by aggregation of the misfolded proteins into insoluble deposits [1,2]. One of the most prominent protein-misfolding disorders is Alzheimer’s disease (AD). It was estimated that 35.6 million people lived with dementia worldwide in 2010, and the number of patients with dementia is expected to reach 65 million by 2030 and 115.4 million by 2050 [3]. There are no effective treatments available for AD. Therefore, much attention is being directed at the development of approaches that counteract the fundamental pathological processes of the disease [4,5]. Several classes of drug candidates that have a potential to be disease-modifying drugs are now under development. However, because of unsatisfactory results, many of these clinical trials have been discontinued [4,6]. Thus, identification of the proper target is of utmost importance for selecting effective AD therapies. There is mounting scientific evidence supporting tau-targeted therapy. (1) Tau neurofibrillary pathology is the major correlate of clinical symptoms in AD [7-9]. (2) Distribution of neurofibrillary pathology defines subtypes of AD with distinct clinical characteristics [10]. (3) Neurofibrillary tangles (NFTs) precede amyloid-β pathology [11-13]. (4) Cortical atrophy measured by magnetic resonance imaging is associated with neurofibrillary pathology [14]. (5) Tau pathology in the absence of amyloid pathology strongly correlates with clinical features in human tauopathies such as progressive supranuclear palsy, corticobasal degeneration, tangle-only dementia, argyrophilic grain disease, frontotemporal dementia and Pick’s disease [15-21]. (6) Tau animal models reproduce neuronal and glial tau pathology leading to the progressive cognitive and/or motor impairment and premature death [5].

The potential of immunotherapeutic strategies to treat AD is one of the most interesting, unexpected and novel findings over the past decade of dementia research. Currently proposed tau immunotherapeutic approaches would target selectively phosphorylated tau species such as phospho-Ser396/phospho-Ser404 [22-24], phospho-Thr231/phospho-Ser235 [25] or abnormally phosphorylated tau species such as phospho-Ser422 [26]. In contrast to studies focused on individual phosphorylated tau species, in our present study we developed an immunoproteomic discovery platform that allowed us to identify immunologically targetable common denominators of pathologically mis-disordered tau species [27] that are responsible for pathological tau–tau interaction and thus for the progress of neurofibrillary pathology. Discovery of these regions on tau is a prerequisite for the development of successful tau vaccines that will protect neurons from pathological tau–tau interaction.

We identified such a common denominator, a domain on mis-disordered truncated tau that is essential for propagation of tau pathology, and we created a monoclonal antibody—DC8E8—that disables this function. DC8E8 prevents tau aggregation *in vitro* and reduces the amount of a wide range of tau oligomers and neurofibrillary pathologies in the brain in transgenic animals. Combined with the ability of DC8E8 to discriminate between healthy and pathological tau with high fidelity, this finding opens a promising avenue to the development of AD treatment.

## Methods

### Ethical approval

All experiments were performed in accordance with the Slovak and European Community Guidelines and with the approval of the Ethics Committee of the Institute of Neuroimmunology, Slovak Academy of Sciences (Bratislava, Slovakia).

### Preparation of hybridoma cell line producing DC8E8

Balb/c mice were immunised with mis-disordered tau protein 151-391/4R. Harvested immune spleen cells were fused with the mouse myeloma cell line NS0 according to a fusion protocol described previously [28]. Growing hybridoma clones were selected for the production of anti-tau-151-391/4R-specific monoclonal antibodies (mAbs) by enzyme-linked immunosorbent assay (ELISA).

### Monoclonal antibodies

The mAbs used in this study are listed in Table 1.

**Table 1:** Antibodies used in this study

**Antibody**	**Epitope**	**Vendor**	**Reference**
DC8E8	tau assembly–regulating domains in MTBR^a^ region	Axon Neuroscience	This study
DC11	truncated mis-disordered tau species	Axon Neuroscience	[27,29]
DC25	tau 347–353	Axon Neuroscience	[30-36]
DC4R	tau 297–305	Axon Neuroscience	[34]
dGAE56	tau 377–384	Axon Neuroscience	
DC144	tau 368–376	Axon Neuroscience	
MN423	Conformational, requiring tau truncated at 391		[37,38]
DC51	Rabies virus		[39]
AT8	tau pS202/pT205	Pierce Biotechnology	
pS214	tau pS214	Invitrogen	

^a^MTBR, Microtubule-binding repeat.

### Screening of monoclonal antibodies using tau–tau interaction assay

The assay to measure the effect of tau-specific antibodies on pathological tau–tau interactions was set up in phosphate-buffered saline (PBS) containing 20 μM (final concentration) of the tested mis-disordered tau protein (151-391/4R), 5 μM heparin (heparin sodium salt from porcine intestinal mucosa, ≥150 IU/mg, dry basis; Sigma-Aldrich, St Louis, MO, USA) and 12.5 μM (final concentration) thioflavin T (Sigma-Aldrich). Each reaction (80 μl final volume) was incubated for 20 hours at 37°C in sealed black solid polystyrene plates (384 wells; Greiner Bio-One, Monroe, NC, USA). Thioflavin T fluorescence was measured using a microplate fluorometer (Fluoroskan Ascent FL; Thermo Labsystems, Milford, MA, USA) with excitation wavelength of 450 nm, emission at 510 nm and 200-ms measurement time. To determine the effect of mAbs on pathological tau–tau interactions, we added purified antibodies at 20 μM final concentrations to the reactions. The reaction mixtures were incubated at 37°C for 20 hours. Antibody DC51 (recognising an envelope protein of the rabies virus [39]) was used as a mock control.

### Western blot analysis of oligomerisation reactions

Mis-disordered tau 297-391/4R was incubated for 1, 4 and 20 hours in either the presence or absence of DC8E8 as described above for the tau–tau interaction assay. At the time points indicated, the reactions were stopped by addition of SDS sample loading buffer. For oligomeric tau analysis, 10 μl of each fibrillisation reaction was electrophoresed and transferred to polyvinylidene fluoride (PVDF) membranes. Subsequently, the membrane was incubated for 1 hour with horseradish peroxidase (HRP)–conjugated DC25 diluted 1:1,000 in PBS. The blot was developed with SuperSignal West Pico Chemiluminescent Substrate (Pierce Biotechnology, Rockford, IL, USA), and the chemiluminescent signals were detected using an LAS3000 imaging system (FUJI Photo Film Co, Tokyo, Japan). The chemiluminescent signal intensities were quantified using AIDA Image Data Analyzer software (Raytest, Straubenhardt, Germany).

### Indirect enzyme-linked immunosorbent assay

Microtitre plates (SARSTEDT, Nümbrecht, Germany) were coated overnight at 37°C with human tau protein isoforms 2N4R, 2N3R, and their deletion mutants, and with tau-derived peptides (250 ng/well). After blocking with 1% fat-free dried milk, the plates were washed with PBS-0.05% Tween 20 and incubated with 50 μl/well of DC8E8 hybridoma culture supernatant for 1 hour at 37°C. Bound DC8E8 was detected by polyclonal goat anti-mouse immunoglobulin (Ig)/HRP antibody (Dako, Carpinteria, CA, USA) using chromogenic substrate *o*-phenylenediamine (Sigma-Aldrich). Absorbance was measured at 492 nm using a Multiskan MCC/340 ELISA plate reader (Thermo Labsystems).

### Competitive enzyme-linked immunosorbent assay

ELISA plates (SARSTEDT) were coated overnight at 37°C with 250 ng/well of mis-disordered purified tau 151-391/4R. Peptide competitors (>95% purity; EZBiolab, Carmel, IN, USA) were dissolved in PBS at a final concentration of 1 mM. A 200 μM solution of peptides in PBS/Tween 20 were filled in wells of polypropylene microtitre plates (Greiner Bio-One). The mAb DC8E8 was diluted to a concentration of 0.6 μg/ml (3.8 nM) in PBS, and 60 μl of the diluted antibody solution was mixed with 40 μl of peptide solution in the polypropylene plate. The antibody/peptide mixtures were incubated for 1 hour at 25°C. Subsequently, 50 μl/well of antibody/peptide mixtures were transferred onto mis-disordered tau 151-391/4R-coated ELISA plates (in duplicates) and incubated for 1 hour at 25°C. Bound DC8E8 was detected using polyclonal goat anti-mouse Ig/HRP (Dako) with the chromogenic substrate *o*-phenylenediamine (Sigma-Aldrich).

### Preparation of DC8E8 antigen-binding fragment crystals

DC8E8 was produced by hybridoma cells in serum-free media. After purification on a 5-ml protein G Sepharose column (GE Healthcare Life Sciences, Pittsburgh, PA, USA), DC8E8 antigen-binding fragment (Fab) was produced by partial digestion with papain (papain from Carica papaya; Roche Diagnostics, Indianapolis, IN, USA) and purified by affinity purification in protein A and protein G media and by size-exclusion chromatography as described previously [40]. The Fab was concentrated to 10 mg/ml in 0.01 M Tris-HCl, pH 7.2, 0.05 M NaCl by ultrafiltration and stored at 4°C. Crystallisation was performed using a vapour diffusion technique at 21°C with 0.5- to 1-μl hanging drops in EasyXtal plates (QIAGEN, Valencia, CA, USA). The drops were prepared by mixing equal volumes of protein and precipitant solution. The final concentration of the Fab was 9 mg/ml. After crystallisation screening using Structure Screen 1 (Molecular Dimensions, Newmarket, UK), diffraction quality crystals were obtained from 0.2 M magnesium chloride, 0.1 M Tris-HCl, pH 8.5, supplemented with 30% wt/vol PEG 4000 (polyethylene glycol; precipitant solution number 33).

### Collection of X-ray diffraction data and data processing

Crystals were mounted in nylon loops (Hampton Research, Aliso Viejo, CA, USA), cryoprotected in Paratone-N (Hampton Research) and flash-cooled by immersion in liquid nitrogen. X-ray diffraction data were collected at - 173°C using synchrotron radiation on the beamline X06DA (Swiss Light Source, Paul Scherrer Institut, Villigen, Switzerland) with a 1.0-Å monochromatic fixed wavelength and a mar225 charge-coupled device X-ray detector (Marresearch/Rayonix, Norderstedt, Germany). A set of 190 images was recorded with a 0.5° oscillation angle, an exposure time of 2.5 seconds per image and a crystal-to-detector distance of 320 mm. Data were indexed, integrated and scaled with *XDS*[41] (30 March 2013 version). The space group was determined using POINTLESS [42]. Unit cell content analysis and data reduction were performed with tools from the CCP4 suite v6.02 [43]. Data collection and processing statistics are reported in Additional file 1.

### Structure solution and refinement

Phases were obtained by molecular replacement with the structure of the 25-D1.16 Fab as a model (Protein Data Bank (PDB) ID 3CVI [44]) using *Phaser* software [45]. Verification of correct packing of the obtained solutions as well as preparation of figures of solved structure was performed using PyMOL (PyMOL Molecular Graphics System, Version 1.5.0.1; Schrödinger, New York, NY, USA). The initial model obtained by molecular replacement was further refined against X-ray data by successive runs of the REFMAC*5* program [46], followed by the manual model adjustments in *Coot* software [47]. The jelly body refinement option of REFMAC*5* with noncrystallographic symmetry (NCS) constraints was used. Following model completion, the NCS constraints were removed. Water molecules were added manually into a positive difference electron density in the *Coot* environment. Because of the missing electron density, the side chains of complementarity determining region (CDR) L1 and of a surface loop in the heavy-chain constant domain were only partially modelled. The progress of refinement was monitored by the drop in the values of R-Work and R-Free parameters and root-mean-square deviation of structure characteristics. The final model was verified using the MolProbity server [48] and was of better quality than 98% of structures with similar resolution. The final model and structure factors were deposited in the PDB under ID 4OZ4. The model contains two independently refined DC8E8 Fab molecules and eight water molecules, which were individually checked for a reasonable electron density and correct hydrogen bonding. Refinement statistics are reported in Additional file 2.

### Affinity and kinetics determination by surface plasmon resonance

A Biacore 3000 instrument with a Sensor Chip CM5 (GE Healthcare Bio-Sciences, Uppsala, Sweden) was used. Amine-coupling reagents (1-ethyl-3-(3-dimethylaminopropyl)carbodiimide, *N*-hydroxysuccinimide, ethanolamine, pH 8.5), P20 detergent and 10 mM sodium acetate pH 5.0 were obtained from GE Healthcare Bio-Sciences. All experiments were performed at 25°C in PBS (pH 7.4) with 0.005% of P20 (PBS-P) as the running buffer. Typically, a quantity of 5,000 response units (RU) of polyclonal anti-mouse antibody (Z0420; DakoCytomation, Glostrup, Denmark) was coupled at pH 5.0 via primary amines simultaneously in two flow cells, one of which was used as a reference in measurement. In each analysis cycle, DC8E8 was captured in the analysed flow cell to reach an immobilisation level of 230 to 250 RU. For the *K*_d_ determination, as well as for the determination of kinetic rate constants, twofold serial dilutions of tau proteins, including PBS-P as a control, were injected at a flow rate 50 μl/min over the Sensor Chip. Kinetic binding data were double-referenced [49] and fitted (using BIAevaluation software version 4.1; GE Healthcare Bio-Sciences) to a two-phase reaction model. Kinetic rate constants were approximated globally, maximal responses were fitted locally and bulk response was set to zero.

### Animal husbandry

Transgenic mice were generated to overexpress mis-disordered tau protein 151-391/3R under the control of the mouse Thy1 promoter. Transgenic animals were born and bred in our animal facility and housed in standard laboratory conditions in plastic cages in a temperature- and humidity-controlled environment with a 12:12-hour light-dark cycle and food and water available *ad libitum*. Efforts were made to minimise the number of animals utilised.

### Vaccine administration

Vaccine was administered once per week when the mice were between 2 and 6 months of age. Purified antibody DC8E8 in PBS was injected intraperitoneally (1 mg of antibody/300-μl dose/animal) into the transgenic mice of the strain R3m/4 expressing human truncated tau. As a control, irrelevant antibody DC51 was injected into R3m/4 transgenic mice following the same vaccination regime and dosing.

### Isolation of soluble tau and sarkosyl-insoluble tau

Brain tissue was homogenized in tenfold weight excess of ice-cold extraction buffer (20 mM Tris, pH 7.4, 800 mM NaCl, 1 mM ethylene glycol tetraacetic acid), 1 mM ethylenediaminetetraacetic acid 0.5% β-mercaptoethanol, 10% sucrose, 1 mM Na_3_VO_4_ and 20 mM NaF, supplemented with a cOmplete Protease Inhibitor Cocktail Tablet (Roche Diagnostics). After incubation on ice for 5 minutes, the homogenates were cleared by centrifugation at 20,000 *g* for 20 minutes at 4°C. The supernatants were collected, and the total protein concentration was determined using a Bio-Rad protein assay (Bio-Rad Laboratories, Hercules, CA, USA). This supernatant (designed 1S) contained soluble tau fraction. Subsequently, solid sarkosyl (*N*-lauroylsarcosine sodium salt; Sigma-Aldrich) was added to the 1S supernatant to achieve 1% concentration and then stirred for 1 hour. Thereafter it was centrifuged at 100,000 *g* for 1.5 hours at room temperature (RT). Following centrifugation, pellets were gently rinsed with 1 ml of the extraction buffer and centrifuged at 100,000 *g* for 20 minutes at RT. The pellets containing sarkosyl-insoluble tau fractions were dissolved in SDS-PAGE loading buffer to a final volume corresponding to the 1/50 volume of the 1S supernatant.

### Immunoblot analysis of soluble tau and sarcosyl-insoluble tau

Samples of sarkosyl-insoluble tau fractions were dissolved in 1× SDS sample loading buffer in a 1/50 volume of the soluble fraction and heated at 95°C for 5 minutes. Each sample (6 μl) was then loaded onto 5–20% gradient SDS polyacrylamide gels and electrophoresed in a Tris-glycine-SDS buffer system for 40 minutes at 25 mA. Proteins were transferred to PVDF membrane (1 hour at 150 mA in 10 mM *N*-cyclohexyl-3-aminopropanesulfonic acid, pH 12), and, after blocking in 5% fat-free dry milk in PBS for 1 hour at room temperature, the membrane was incubated for 1 hour with pan-tau mAb DC25. After washes, HRP-conjugated goat anti-mouse Ig (Dako Denmark) diluted 1:4,000 in PBS was used as a secondary antibody. Incubation (1 hour at room temperature) was followed by washing with 0.2% IGEPAL (Sigma-Aldrich) in PBS. The blots were developed with SuperSignal West Pico Chemiluminescent Substrate (Pierce Biotechnology), and the signal was detected using the LAS3000 imaging system. The signal intensities were quantified using AIDA software and then statistically evaluated using an unpaired *t*-test.

### Immunohistochemistry of mouse brain tissue samples

Transgenic mice were deeply anaesthetised with Zoletil-xylazine and perfused intracardially using a peristaltic pump for 2 minutes with PBS. The brain was postfixed overnight in 4% paraformaldehyde in PBS, pH 7.2, cryoprotected with 15% and 25% sucrose solutions (subsequently overnight), frozen in 2-methylbutane (30 seconds at −42°C) and transferred to dry ice. Sagittal brainstem sections (40 μm thick) were cut on a Leica CM1850 cryomicrotome (Leica Biosystems, Buffalo Grove, IL, USA). For semiquantitative analysis, two sections were quantified. Tissue sections were incubated with primary antibodies AT8 (Pierce Endogen) and pS214 (Invitrogen, Carlsbad, CA, USA) overnight at 4°C. Sections were immunostained using the standard avidin-biotin-peroxidase method (VECTASTAIN Elite ABC Kit; Vector Laboratories, Burlingame, CA, USA) with VIP as the chromogen.

### Immunohistochemistry of human brain tissue samples

Human brain tissue samples were obtained from Netherlands Brain Bank in accordance with local ethical approval and written consent from the donors or their next of kin. Ethical approval was obtained for the analyses carried out using this tissue (Institute of Neuroimmunology, Slovak Academy of Sciences, 5/2011). Immunohistochemical staining was performed on paraffin sections after deparaffinisation. The brain sections were treated with 99% formic acid for 1 minute and then in a pressure cooker for 20 minutes. Afterwards, sections were incubated for 20 minutes at room temperature in 1% H_2_O_2_, followed by a 30-minute incubation in blocking solution (0.01 M PBS containing 0.3% Triton X-100 and 1% horse serum). This was followed by overnight incubation at 4°C in primary antibody DC8E8 in blocking solution. After washing, the sections were incubated in biotinylated secondary antibody (VECTASTAIN; Vector Laboratories). The reaction product was visualised using avidin-biotin and Vector VIP as the chromogen (Vector Laboratories).

### Statistical analysis

Statistical analysis was carried out using the Prism statistical software package (GraphPad Software, La Jolla, CA, USA). To compare two groups, the Mann–Whitney *U* test or an unpaired *t*-test was applied. The results are presented as mean ± standard error of the mean unless otherwise specified. Differences were considered significant at the level of *P* < 0.05.

## Results

### DC8E8 candidate therapeutic antibody is inhibiting pathological tau–tau interactions

**Figure 1:**
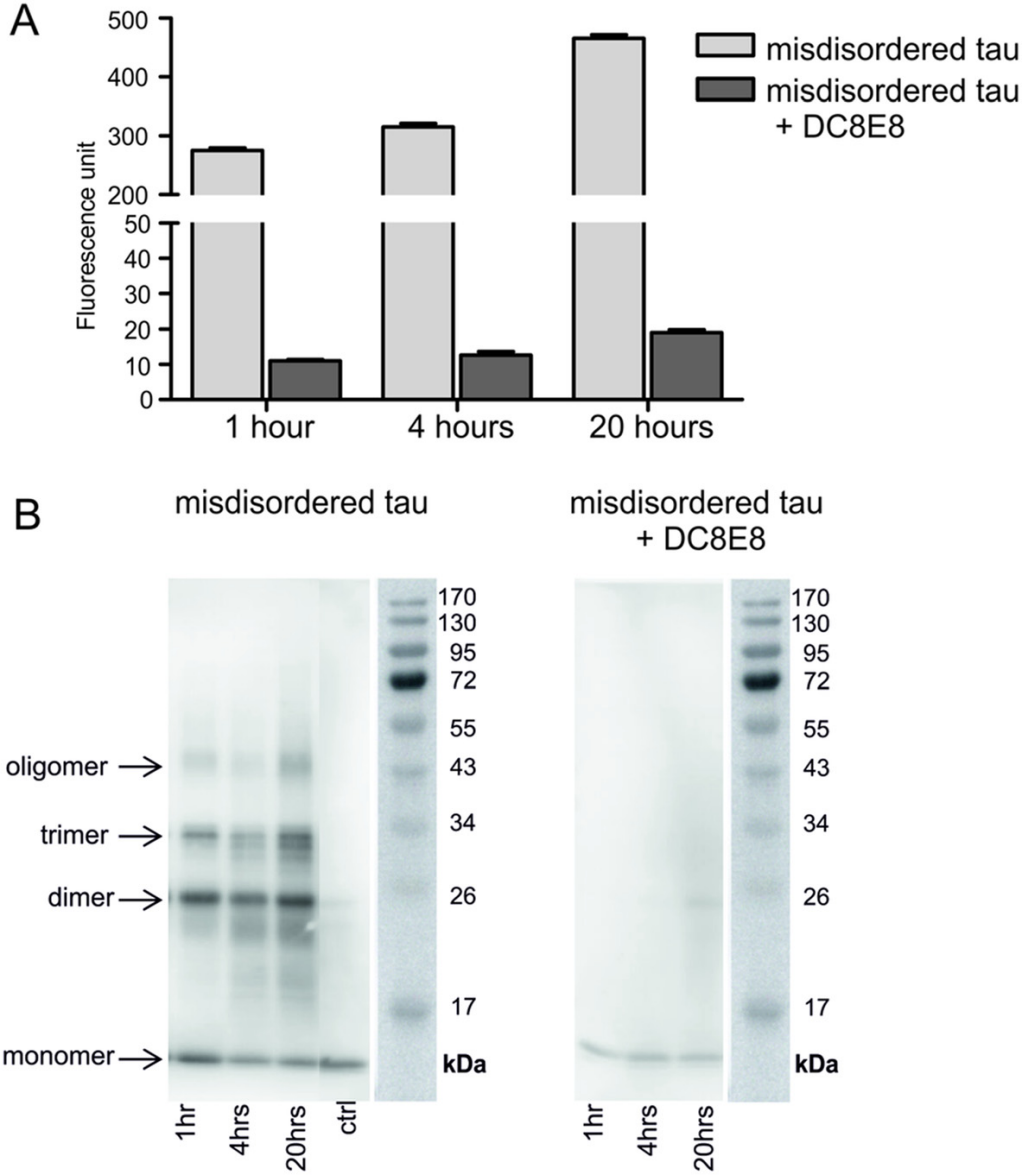
**Monoclonal antibody DC8E8 inhibits pathological tau–tau interaction. (A)** Inhibition of pathological tau–tau interaction by DC8E8. The amount of oligomerised tau (297-391/4R) was measured by thioflavin T fluorescence in the absence and in the presence of the monoclonal antibody (mAb) DC8E8 at the time points 1, 4 and 20 hours. Inhibitory activity of DC8E8 was statistically significant for the indicated time points when analysed using a nonparametric *t*-test (*P* < 0.0001). **(B)** Analysis of the inhibitory potential of DC8E8 showing prevention of the formation of tau dimers, trimers and oligomers by mis-disordered truncated tau by immunoblotting using horseradish peroxidase–conjugated mAb DC25. The positions of the molecular weight markers are indicated on the right.

In order to identify immunologically targetable structures on tau with therapeutic potential, we used an *in vitro* pathological tau–tau interaction assay to screen for mAbs with an inhibitory effect on pathological tau–tau interactions. For this screening, a panel of tau protein–specific antibodies was used (Table 2). The amount of conformationally altered and oligomerised tau was measured by thioflavin T fluorescence in the absence and in the presence of the respective tested antibody. The screening revealed that the mAb DC8E8 was most efficient in prevention of the pathological conformational change and fibrillisation of tau proteins compared with other tested tau-specific antibodies (Table 2). The antibody reduced the amount of oligomerised truncated tau by 84% when measured by thioflavin T fluorescence (Table 2). Surprisingly, within the panel of screened antibodies targeting mis-disordered tau, some antibodies displayed an opposite effect, that is, not inhibition but enhancement of tau oligomerisation (Table 2, mAb MN423 and DC11). A mock antibody, DC51 [39], which does not bind to tau, did not influence the conformational change of tau, resulting in unaltered thioflavin T fluorescence. DC8E8 inhibitory activity on tau oligomerisation was examined at the various time points (1, 4 and 20 hours) (Figure 1A) and was highly statistically significant when analysed using an unpaired *t*-test (*P* < 0.0001). Furthermore, oligomerisation reactions from tested time points were analysed using immunoblotting as well. The analysis showed that DC8E8 inhibited the whole process of tau oligomerisation, that is, the formation of tau dimers, trimers and higher-order oligomers of mis-disordered tau (Figure 1B). These results reveal that the binding site recognised by DC8E8 is essential for pathological transition of tau leading through oligomerisation to mature forms of tau NFTs.

**Table 2:** **Screening of monoclonal antibodies that inhibit pathological tau–tau interaction revealed DC8E8 as the best candidate**^**a**^

**Antibody**	**Inhibition of tau oligomer formation (%)**
DC8E8	84
DC4R	25
dGAE56	16
DC25	12
DC144	−14
DC11	−45
MN423	−60
DC51	0

^a^The oligomerisation of misfolded truncated tau (151-391/4R) was measured by thioflavin T fluorescence. Monoclonal antibodies were tested for their ability to prevent the pathological tau–tau interactions. DC8E8 showed the highest potency of inhibition of tau oligomerisation. Monoclonal antibody DC51 recognising an envelope protein of rabies virus was used as a mock control. For a detailed characterisation of antibodies, see Table 1.

### DC8E8 recognises neurofibrillary pathology in preclinical, clinically incipient and fully developed Alzheimer’s disease

**Figure 2:**
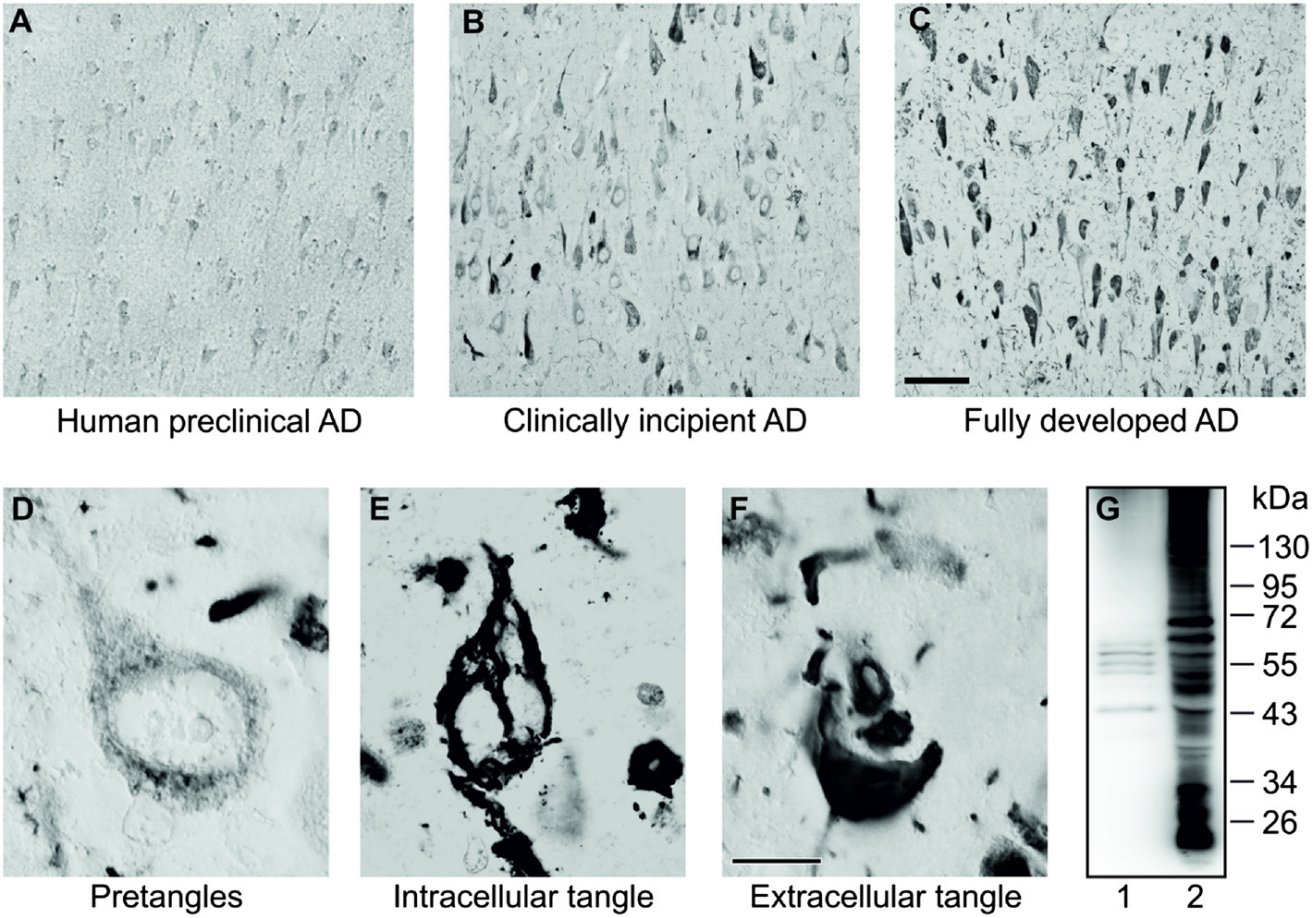
**Monoclonal antibody DC8E8 recognises developmental stages of Alzheimer’s disease tau neurodegeneration and Alzheimer’s disease–specific insoluble tau complexes.** DC8E8 recognises preclinical Alzheimer’s disease (AD) **(A)**, clinically incipient AD **(B)** and the fully developed final stage of AD **(C)**. Hippocampal staining shows that DC8E8 detects early pretangle stages **(D)**, intracellular neurofibrillary tangles **(E)** and extracellular neurofibrillary tangles **(F)**. Tool bar: A-C 100 µm; D-F 10 µm. **(G)** DC8E8 recognizes soluble tau (*lane 1*) and sarkosyl-insoluble tau proteins (*lane 2*) in the material extracted from Braak stage VI AD brain tissue (allocortex tissue including hippocampus, entorhinal and temporal cortex). Western blot shows that DC8E8 detects AD-specific tau species with low and high molecular weights. For soluble tau (*lane 1*), 15 μg of total protein was loaded per lane. Sarkosyl-insoluble tau fraction (*lane 2*) was 50-fold enriched by solubilisation in a small volume of SDS-PAGE loading buffer (for details, see the Methods section).

Immunohistochemical analysis showed that DC8E8 enabled us to detect early stages of pathological tau in human preclinical AD (Braak stage I) (Figure 2A). At this stage, tissue contains only a limited number of NFTs in the entorhinal cortex and no NFTs in the hippocampus. In the clinically incipient AD brain, where a few NFTs were found in the hippocampus (Braak stage III), the DC8E8 mAb recognised both the stage of pathological tau oligomers and the stage of pathological tau polymers (tangles) (Figure 2B). In AD brain with fully developed neurofibrillary degeneration, DC8E8 recognised mainly pathological tau polymers in the form of NFTs, neuritic plaques and neuropil threads (Figure 2C). DC8E8 targets all developmental stages of neurofibrillary lesions in human AD brain tissue, including pretangle stage (Figure 2D), intracellular NFTs (Figure 2E) and extracellular NFTs (Figure 2F).On the molecular level, DC8E8 recognises truncated tau proteins, full-length tau proteins corresponding to monomeric tau forms and assembled tau proteins with higher molecular weights corresponding to oligomeric tau forms in the human brain tissue assessed by Western blot analysis (Figure 2G).

### DC8E8 immunotherapy significantly reduces levels of insoluble tau oligomers in transgenic mouse brain

**Figure 3:**
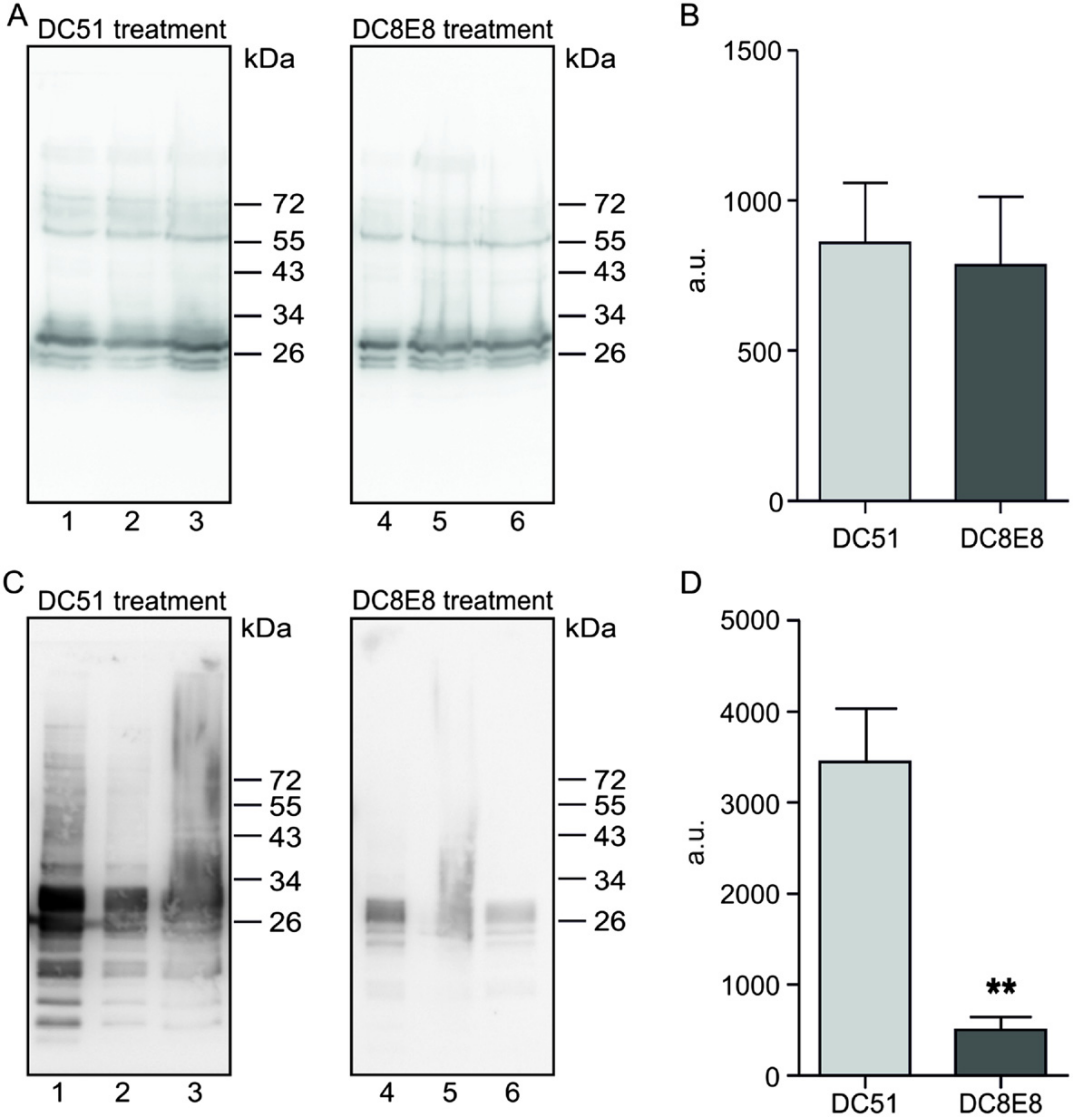
**DC8E8 immunotherapy significantly reduces levels of insoluble tau oligomers in brains of transgenic mice.** Tau transgenic mice were immunized with DC8E8 and with an irrelevant antibody, DC51, and brain tissue of the animals was fractionated into soluble and sarcosyl-insoluble tau fractions. **(A)** and **(B)** Amount of total soluble tau is unchanged after DC8E8 treatment compared with treatment with a mock antibody, DC51. **(C)** and **(D)** Amount of sarcosyl-insoluble tau oligomers in transgenic mouse brain is significantly diminished after treatment with DC8E8 (*n* = 3, **P < 0.01). All Western blots were stained with pan-tau monoclonal antibody DC25, and lanes 1 to 6 contain samples from individual animals used in the experiments. Signal quantification was performed using AIDA software.

In order to evaluate its therapeutic activity, we administered the candidate therapeutic antibody DC8E8 and mock antibody DC51 to transgenic mice, line R3m/4, expressing mis-disordered human tau under the Thy1 promoter. The transgenic mice show an age-dependent development of NFTs and formation of sarcosyl-insoluble tau oligomers in the brainstem that are associated with the onset of progressive motor dysfunction leading to premature death at 6 to 7 months of age. Transgenic mice received injections first at the age of 2 months and then once per week for 4 months. The mice were killed 1 week after the final dose. We found that DC8E8 therapy significantly reduced the levels of sarcosyl-insoluble tau oligomers (89%) (Figure 3C and D). Strikingly, total tau levels did not differ between the groups (Figure 3A and B).

### DC8E8 immunotherapy decreases load of neurofibrillary tangles in brains of transgenic mice expressing mis-disordered tau

**Figure 4:**
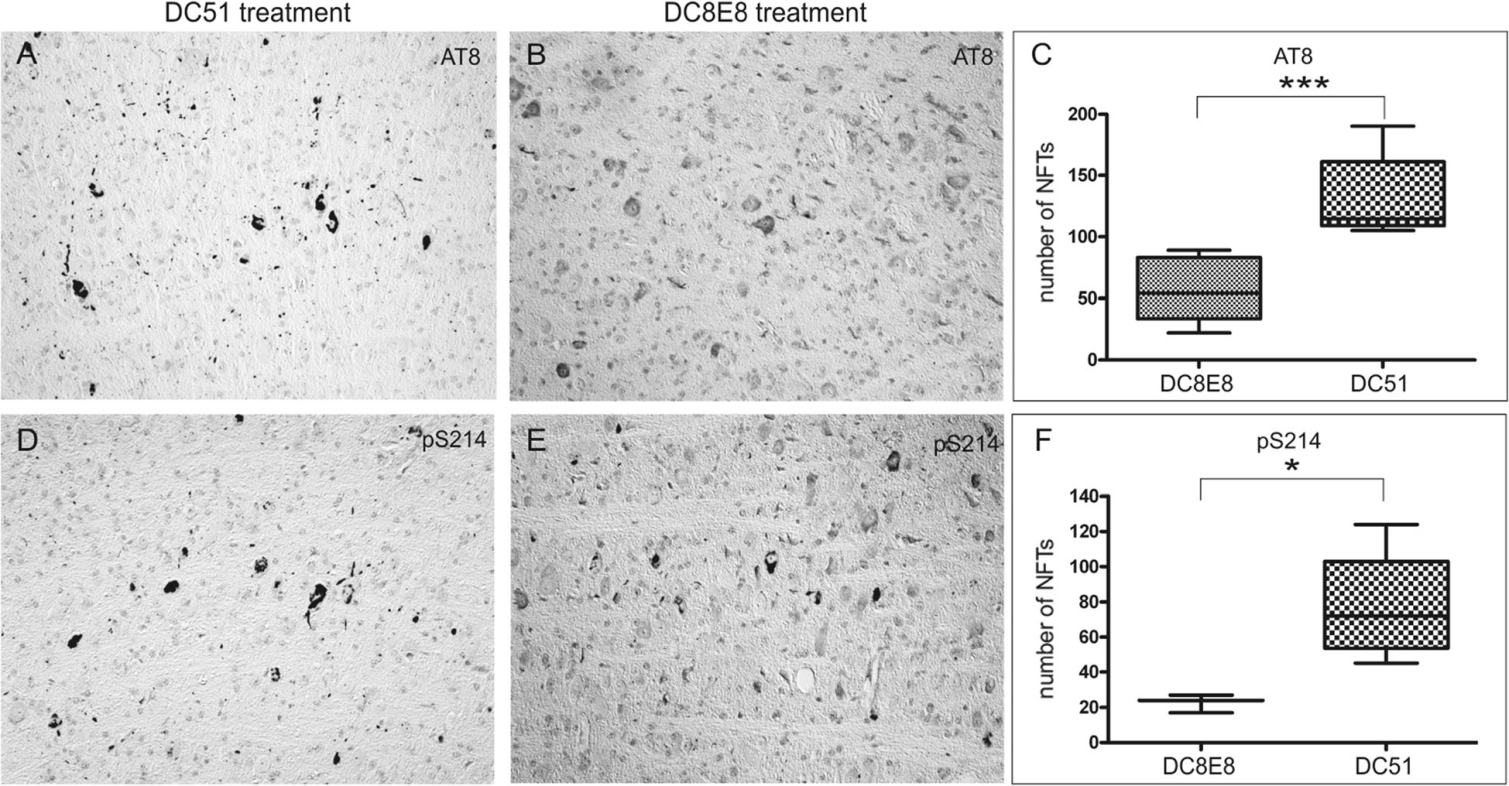
**DC8E8 significantly reduces the number of neurofibrillary tangles in transgenic mice.** Transgenic mice were treated with mock antibody DC51 **(A)** and **(D)** and with DC8E8 **(B)** and **(E)**. Neurofibrillary tangles (NFTs) were visualised with AT8 staining **(A)** and **(B)** and with pS214 antibody **(D)** and **(E)**. Transgenic mice treated with therapeutic antibody DC8E8 showed significantly less tau pathology than mice treated with mock antibody DC51 **(C)** and **(F)**. * - P < 0.05; *** - P < 0.001; boxes represent 75 percentiles, middle bars represent medians and outer horizontal bars represents data range.

To assess the impact of the DC8E8 immunotherapy on the tangle load we carried out an immunohistochemical analysis using two phosphorylation dependent antibodies. Monoclonal antibody AT8 (pSer202/pThr205) recognises all stages of tangle formation [30,50-53] and antibody pS214 (pSer214) stains predominantly late stages of tangle formation. Treatment of transgenic mice with mock antibody DC51 had no impact on the load of NFTs (Figure 4A and D). However, immunotherapy of transgenic mice with candidate therapeutic antibody DC8E8 significantly reduced early stages (Figure 4B and C) (*P* < 0.001) and late stages of tangle formation (*P* < 0.05) (Figure 4E and F) compared to those treated with mock antibody.

### DC8E8 targets four separate structural determinants essential for pathological tau–tau interaction

**Figure 5:**
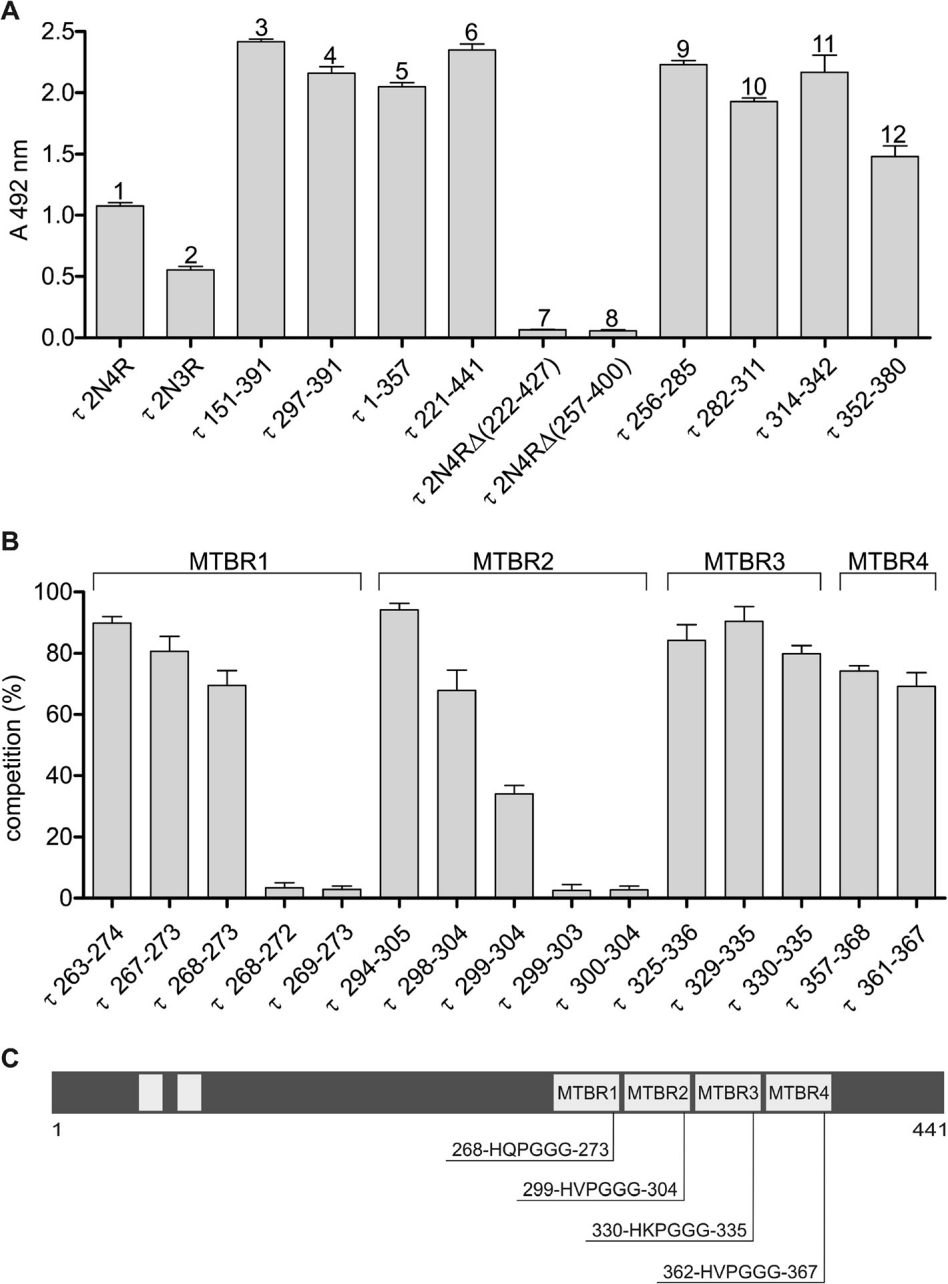
**Mapping of domain on tau protein recognised by DC8E8. (A)** For the epitope mapping of DC8E8 monoclonal antibody, we used full-length three- and four-repeat tau isoforms with two N-terminal inserts (*1* and *2*), tau deletion mutants (*3*–*8*) and tau-derived synthetic peptides (*9*–*12*). All deletion mutants that contained the microtubule-binding repeat (MTBR) region (*3*–*6*) were recognized by DC8E8; tau deletion mutants lacking the MTBR region were not recognized (*8* and *9*). Importantly, DC8E8 recognized each of synthetic peptides derived from individual MTBRs (*9*–*12*). The result is that DC8E8 recognised four binding sites (epitopes) located in the MTBR region of the tau protein, and each epitope is separately located within one MTBR. **(B)** To narrow down the DC8E8 minimal epitope, homologous peptides derived from the tau protein repeat region (MTBR1–4) were analysed in competitive enzyme-linked immunosorbent assays. Tau peptides containing at least six amino acids of the DC8E8 recognition sequence HXPGGG were capable of competing with mis-disordered tau (amino acids 151–391) for binding to antibody DC8E8. Tau peptides containing five amino acids of the DC8E8 recognition sequence did not compete with mis-disordered tau for binding to antibody DC8E8. **(C)** Schema of epitopes on tau protein molecule recognised by DC8E8. The DC8E8 monoclonal antibody is capable of binding four separate binding regions, with each region forming one individual epitope. These four epitopes are separately located within the first, second, third and fourth MTBR of protein tau. *Note*: All listed molecules are numbered in reference to the longest human tau isoform (2N4R) sequence.

Epitope mapping of DC8E8 using deletion mutants of human tau protein 2N4R, as well as mapping using tau-derived synthetic peptides, suggested that the DC8E8 binding site is located in the microtubule-binding repeat (MTBR) region of the tau protein. Moreover, this analysis revealed that DC8E8 binds four structural determinants, epitopes, on human tau, each of which is separately located within one MTBR (Figure 5A). Furthermore, DC8E8 displays a significant preference for mis-disordered tau protein deletion mutants (151-391/4R, 297-391/4R, 1-357/4R and 221-441/4R) over the physiological full-length tau isoforms 2N4R and 2N3R (Figure 5A). DC8E8 did not recognise tau constructs where the MTBR region was deleted (tau 2N4RΔ(222-427) and tau 2N4RΔ(257-400)).

To define the minimal epitopes recognised by DC8E8, additional tau peptides (5-mers, 6-mers, 7-mers and 12-mers) derived from each MTBR were designed. All peptides were analysed for their ability to compete with mis-disordered tau (151-391/4R) for binding to DC8E8. As shown in Figure 5B, 6-mer peptides derived from four MTBRs (tau 268–273 from MTBR1, tau 299–304 from MTBR2 identical to tau 362–367 from MTBR4 and tau 330–335 from MTBR3) were able to compete with mis-disordered tau (151-391/4R) for binding to DC8E8. However, removal of either N-terminal histidine (5-mer peptides; tau 269–273, tau 300–304) or the C-terminal glycine (5-mer peptides, tau 268–272, tau 299–303) abolished competition of these peptides with tau (151-391/4R) for binding to DC8E8 (Figure 5B). This shows that the 6-mer peptides with consensus sequence HXPGGG localised in the MTBR region of tau are sufficient for DC8E8 recognition. At the same time, peptides six amino acid residues long encompassing the consensus sequence HXPGGG are necessary to create the three dimensional structure recognized by DC8E8. Thus, we can draw three conclusions. (1) The HXPGGG amino acid sequence represents the minimal epitope, structural determinant on mis-disordered protein tau recognised by DC8E8. (2) The minimal DC8E8 epitope on mis-disordered human tau is present four times as four separate structural determinants, epitopes, each encompassing six amino acids. (3) The primary structure of epitope 1 is ^268^HQPGGG^273^ (located within MTBR1), that of epitope 2 is ^299^HVPGGG^304^ (located within MTBR2), that of epitope 3 is ^330^HKPGGG^335^ (located within MTBR3) and that of epitope 4 is ^362^HVPGGG^367^ (located within MTBR4) (Figure 5C).

### Atomic structure of DC8E8 binding site recognizing determinants of pathological tau–tau interaction

**Figure 6:**
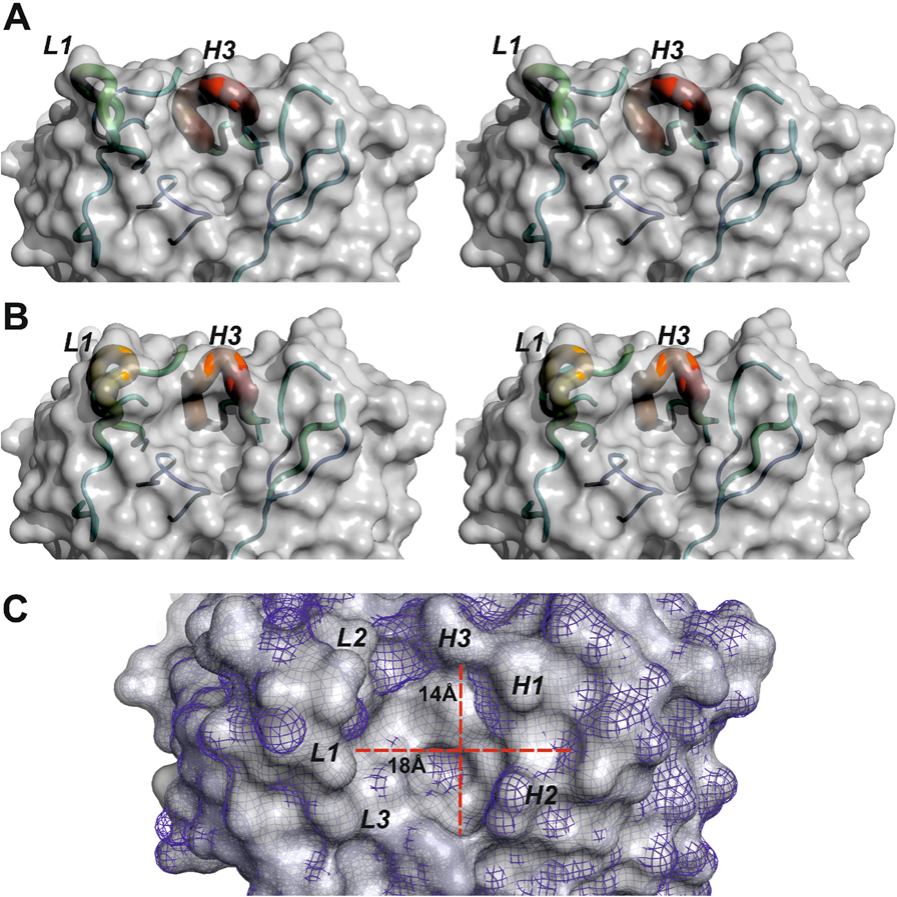
**The flexibility of the binding site allows DC8E8 to adapt to four homologous, albeit not identical, structural determinants in the tau microtubule-binding repeats.** Two independently refined X-ray structures of DC8E8 antigen-binding fragment **(A)** and **(B)** (stereoview) show the flexibility of the antigen-binding site. The surface of the antibody is shown in grey. The backbones of complementarity determining regions (CDRs) are represented as tubes, with the diameter and colour reflecting their averaged atomic displacement parameters, that is, flexibility. The flexibility is expressed as a colour scale ranging from blue to red, corresponding to B-factors 30 to 150 Å^2^. The CDRs L1 and H3 exhibit higher B-factors than the remaining parts of the model. The pronounced flexibility of these CDRs is essential to allowing DC8E8 to bind each of four slightly different epitopes within the microtubule-binding repeats (MTBRs). **(C)** Superposition of both independently refined DC8E8 molecules shows that the core of the binding pockets is invariant (molecule A shown as grey solid, molecule B as blue mesh). The CDR loops (italic letters) create a 7- to 9-Å-deep pocket with surface dimensions 18 × 14 Å (red axes). The shape of this pocket necessitates that the minimal DC8E8 epitope HXPGGG adopts a fold protruding into this space to bind in the DC8E8 combining site.

In order to better understand the mode of DC8E8 recognition of strategic tau epitope structure (the regulatory domain for pathological tau–tau interactions), we determined the structure of the DC8E8 binding site by X-ray crystallography. DC8E8 crystallised in a monoclinic P21 space group, with two molecules of the Fab in the asymmetric unit (AU). The structure was solved by molecular replacement and refined to 3.0-Å resolution. Both molecules of the Fab in the AU were refined independently in order to allow assessment of the flexibility of the DC8E8 binding site. The backbone of all CDR loops can be traced into the map of electron density. CDR L1 and H3 are partially disordered, as reflected by a weak electron density and high atomic displacement parameters (B-factors; Figure 6A and B). The flexibility of DC8E8 CDR loops appears to be important for primary contact and recognition of the DC8E8 epitope on the tau molecule. The flexibility of the DC8E8 binding site explains the independent recognition of each of the four homologous, albeit not identical, DC8E8 structural determinants in the tau MTBR1-4.The general shape of the DC8E8 binding pocket is similar in both independently refined Fab molecules. Six CDR loops form a relatively deep binding cleft (7 to 9 Å) with well-defined walls and surface dimensions 18 × 14 Å (Figure 6C). It suggests that the minimal DC8E8 tau protein epitope HXPGGG identified by ELISA forms a sharply protruding turn in the complex with antibody or, most importantly, even in the unbound state in the solution.

### DC8E8 kinetically prefers interaction with truncated tau proteins

**Figure 7:**
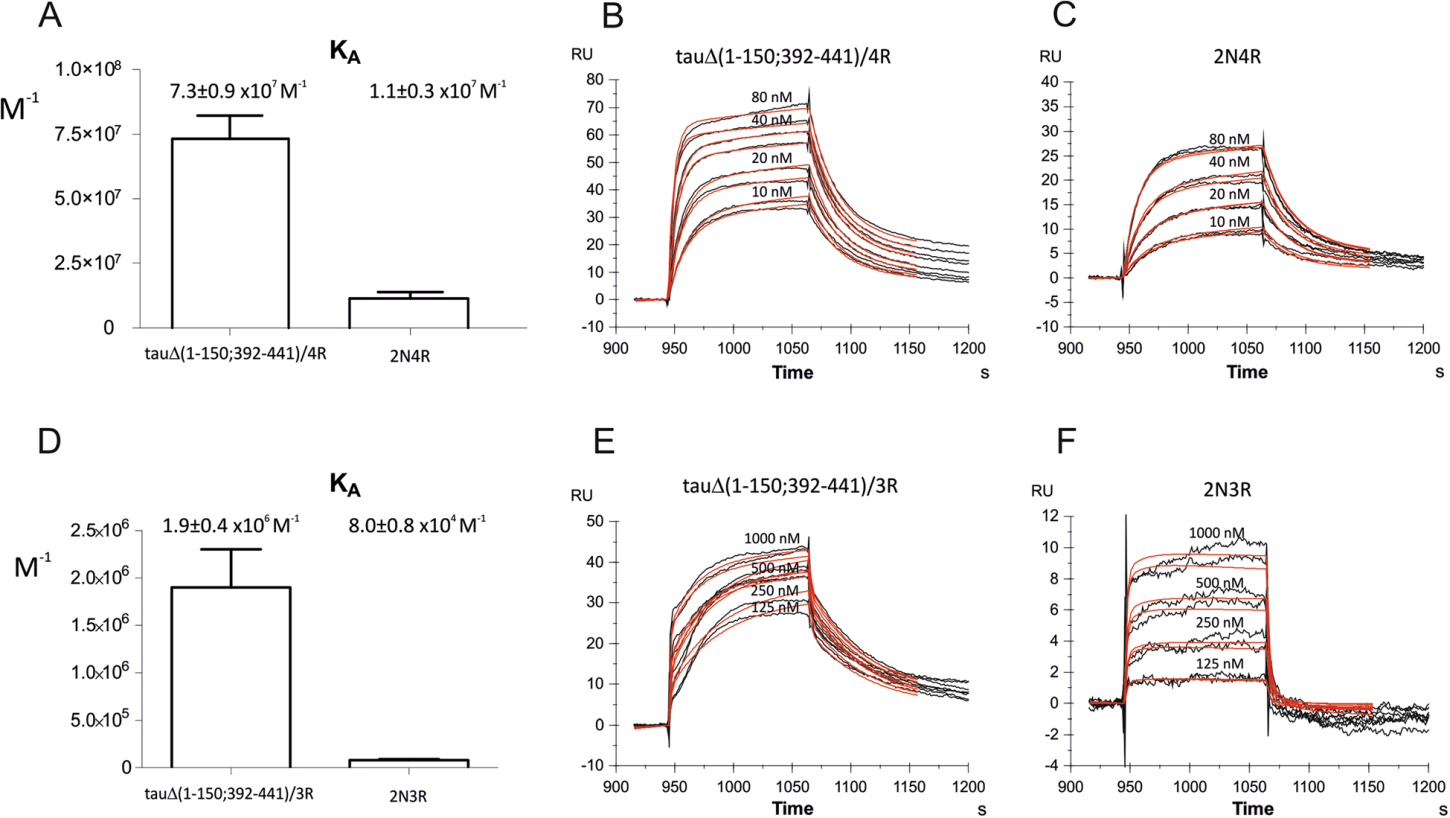
**DC8E8 discriminates between pathological and physiological ‘healthy’ tau. (A)** Affinity comparison of DC8E8–four-repeat tau protein complex formation. The monoclonal antibody DC8E8 exhibits preferential affinity to the mis-disordered truncated tau 151-391/4R. Surface plasmon resonance (SPR) revealed that mis-disordered truncated tau protein is recognised by DC8E8 nearly seven times stronger than the full-length tau protein isoform. Extrapolation of kinetic SPR sensorgrams of DC8E8 interaction with mis-disordered truncated **(B)** and full-length **(C)** four-repeat tau proteins revealed that mis-disordered truncated tau is recognised by DC8E8 with *k*_on_ = 2.9 × 10^6^ M^−1^ s^−1^ and *k*_off_ = 0.04 s^−1^, whereas full-length tau exhibits *k*_on_ = 4.4 × 10^5^ M^−1^ s^−1^ and *k*_off_ = 0.04 s^−1^. **(D)** DC8E8 affinity comparison of three-repeat truncated tau and its full-length counterpart. Similarly to the four-repeat tau, extrapolation of kinetic SPR sensorgrams of DC8E8 interaction with truncated **(E)** and full-length **(F)** three-repeat tau proteins revealed that truncated tau is recognised by DC8E8 with *k*_on_ = 1.5 × 10^5^ M^−1^ s^−1^ and *k*_off_ = 0.08 s^−1^, whereas full-length tau exhibits *k*_on_ = 2.8 × 10^4^ M^−1^ s^−1^ and *k*_off_ = 0.4 s^−1^. These results show that three-repeat mis-disordered truncated tau protein is recognised by DC8E8 with 25 times higher affinity than full-length three-repeat tau protein. Black curves represent experimental data, and red curves were fitted by evaluation software for kinetic parameter calculations.

Surface plasmon resonance (SPR) detection of protein binding is able to determine the kinetic and thermodynamic parameters of protein complexes by direct monitoring of the binding event in real time. In order to further examine the specific way in which DC8E8 antibody recognises tau protein epitopes, we employed SPR to follow interactions of DC8E8 with four- and three-repeat tau protein isoforms and their truncated variants. Determination of the association constant of DC8E8 binding to the four repeat tau protein isoform 2N4R and mis-disordered tau 151-391/4R showed seven times higher affinity for mis-disordered tau than for the full-length tau isoform 2N4R (Figure 7A). We observed an even greater (25 times) selectivity of recognition between mis-disordered tau 151-391/3R and three-repeat tau protein isoform 2N3R (Figure 7B). These results confirm the specificity of DC8E8 to the pathologically truncated mis-disordered form of tau and the selective recognition of mis-disordered tau over the physiological tau. Further, mAb DC8E8 exhibited nearly six times faster binding (higher *k*_on_) to the mis-disordered tau than to the full-length tau (Figure 7B and C). Higher *k*_on_ means greater accessibility of DC8E8 epitope in the frame of mis-disordered tau in comparison to the full-length isoform. The same pattern of preferred recognition of mis-disordered tau protein over the full-length variant was observed for the three-repeat tau protein (Figure 7E and F). Thus, DC8E8 is highly discriminatory between physiological and mis-disordered tau. Higher accessibility of DC8E8 epitopes in the tubulin-binding region of truncated mis-disordered tau proteins can be a direct consequence of a missing C terminus in truncated tau proteins. Indeed, the C-terminal domain of tau was previously suggested to transiently fold back in a hairpinlike fashion onto the repeats of the full-length tau molecule [54], and the removal of C-terminus can increase the accessibility of repeat regions in the mis-disordered tau.

## Discussion

AD is the leading cause of senile dementia. Its prevalence is predicted to increase at least threefold between 2000 and 2050, rendering AD an increasing worldwide public health problem [55]. The current most effective treatment approach for AD—cholinesterase inhibitors and *N*-methyl-D-aspartate receptor antagonists—is purely symptomatic and provides benefit for only up to 12 months [6]. Therefore, the aim of the currently proposed therapeutic approaches and strategies for AD is to counteract the fundamental underlying pathological processes leading to the development and progression of the disease.

The main neuropathological hallmarks of AD are NFTs composed of tau protein and senile plaques consisting of amyloid-β. It has been shown that diseased modified tau proteins play an essential role in the clinical manifestation of AD [7,9,56]. An increased understanding of the molecular mechanisms underlying the pathological transformations of tau has opened up the possibility of specifically targeting pathologically modified tau protein for therapeutic purposes. As a result, a number of therapeutic approaches that directly or indirectly target the tau misfolding cascade have emerged [2,57,58]. One of these promising therapeutic approaches is immunotherapy targeting various tau species. Several independent studies have shown that either active or passive immunotherapy could prevent tau aggregation or clear tau aggregates and reduce tau hyperphosphorylation [22-24,26,59-62]. However, it has been discussed that phosphorylated tau antigens, used predominantly in the abovementioned studies, display some potential risks, as these phosphorylation sites are associated mainly with matured NFTs and not with early stages of tangle formation [63]. Moreover, phosphorylation is the main physiological mechanism regulating tau structure and function, and, therefore, the major concern caused by active immunisation with phospho-tau peptides is an immune response towards the physiological tau species [25].

In this report, we present a preclinical immunoproteomic platform for the identification of structural determinants on tau protein essential for its pathological interaction. Our strategy was based on our findings that truncation of tau protein significantly changes the structure and function of the molecule. Thus, mis-disordered truncated tau is a substrate for pathological tau–tau interactions [27,30,31]. To identify the critical structural determinants on tau responsible for pathological tau–tau interaction, we developed molecular imprinting technology based on the unique properties of mAbs recognizing structural changes of naturally disordered proteins. Using hypothesis-driven research approaches, we proposed a targeted vaccine development strategy consisting of the following steps: (1) identification of a mAb that is able to prevent tau from its pathological tau–tau interaction, (2) *in vivo* validation of the therapeutic activity of the antibody with special emphasis on reduction of neurofibrillary pathology and sarcosyl-insoluble tau, (3) identification of the epitopes on tau recognised by therapeutic antibody, (4) identification of the three-dimensional structure of these binding sites and (5) characterisation of the antibody’s ability to discriminate between disease-modified and physiological tau protein. The chosen algorithm allows us to identify structural determinants essential for pathological tau–tau interaction and validate it as a novel therapeutic target for tau immunotherapy in AD.

In order to identify an antibody that can protect tau from pathological tau assembly, we have developed an *in vitro* assay for screening of antibody potency to inhibit pathological tau–tau interaction. Previously, it has been shown that the key regions responsible for tau–tau interactions are located in the microtubule-binding domain of tau [37,64,65]; however, their pathologic gain of function is regulated by the truncation of distant parts of the tau protein molecule [27]. The phenomenon of regulation of protein function by truncation is particularly prominent in the intrinsically disordered proteins involved in neurodegenerative diseases [66]. It has been shown that tau truncation is one of the key pathognomonic features of neurodegenerative processes in AD [32]. Furthermore, removal of tau protein termini triggers a tau misfolding cascade and the development of tau neurofibrillary pathology in animal models [30,31]. In our present study, we found truncated tau protein 151-391/4R to be particularly suitable for screening for antibodies inhibiting pathological tau–tau interaction. It allowed us to identify DC8E8 antibody as extremely efficient in inhibiting tau–tau interaction. Proteomic mapping revealed that the antibody was capable of binding to four independent and yet highly homologous binding regions, each of which is a separate epitope, one in each of the MTBR regions. DC8E8 represents the first specific antibody recognizing four previously unidentified functional regions of tau (structural determinants, epitopes) regulating pathological tau–tau interaction.

In line with this, DC8E8 inhibits the formation of dimers, trimers and oligomers by mis-disordered tau. Therefore, identified DC8E8 epitopes are involved in tau conformational changes leading to tau oligomerisation as the first step of tau fibrillisation. As DC8E8 is capable of discriminating between pathological and physiological tau proteins, at least one of these four epitopes is conformational.

It is probable that binding of DC8E8 to physiological tau at one or more of these epitopes impedes in the same manner certain conformational changes in the tau that are needed for the oligomerisation of tau. In other words, targeting of these epitopes by antibody influences the structure of adjacent regions with β-structural propensities (for example, 274–281, 306–311 [65]). Thus, binding of DC8E8 to one of these four epitopes within normal tau is capable of preventing one of the earliest pathological changes in tau, connected with the formation of β-sheets within tau. All of the four epitopes targeted by DC8E8 are present in the sequence of the AD paired helical filament (PHF) core [37]. Inhibition of these motifs by DC8E8 and subsequent prevention of PHF core formation is a good explanation for the mechanism underlying how DC8E8 is capable of interfering with the multiple tau-mediated activities contributing to AD pathology, including (1) transition from physiological tau to pathological tau; (2) formation of tau dimers, trimers and other tau oligomers; and (3) formation of insoluble tau aggregates.

We have determined the X-ray structure of a DC8E8 binding site revealing a relatively flexible, deep binding pocket. The pronounced flexibility of CDR L1 and CDR H3 (Figure 6A and B) can facilitate recognition of four independent homologous epitopes on tau. The topology of the DC8E8 binding pocket indicates a protruding shape adopted by the HXPGGG epitope in the tau–DC8E8 complex, suggesting a 180° turn on the tau chain. As we have shown that DC8E8 targets the regulatory motif of tau–tau interaction, it is likely that formation of such a protruding turn by the HXPGGG tau sequence and binding of this turn by DC8E8 efficiently blocks interaction of downstream aggregation-prone tau motifs [37,64,65].

We have proven the antibody’s therapeutic efficacy in an *in vivo* model. To date, tau-targeted immunisation has been explored exclusively in different mutant tau mouse models because these mice develop NFTs in their brains [5]. However, no tau mutations have thus far been observed in AD. Tau protein mutations on chromosome 17 correlate with cognitive and motor impairment in frontotemporal dementia linked to chromosome 17 [67,68]. It is important to note, however, that posttranslational modifications of tau protein, such as truncation [37,69,70], abnormal hyperphosphorylation [71], glycosylation [72], glycation [73,74], ubiquitination [75], polyamination [76] and nitration [77], play key roles in tangle development. They can significantly influence conformational characteristics of tau and impose novel toxic properties onto mis-disordered tau. Thus, in order to test the biological function of the inhibitory antibody DC8E8, we used an animal model of AD based on an AD-relevant disease modification of tau—truncation. Our transgenic mice of the strain R3m/4 express truncated tau derived from human AD brains and display early onset of AD tau pathology, which renders this model an ideal test system for immunotherapy targeting tau neurofibrillary lesions. We treated transgenic animals with DC8E8 for 4 months starting at their second month of life. The antibody was able to significantly reduce early and late tau pathology, showing that DC8E8 targets and disables disease-modified mis-disordered tau. Importantly, antibody DC8E8 recognises all forms of tau lesions, including pretangles and intracellular and extracellular NFTs, in both preclinical and fully developed human AD. Therefore, it is reasonable to expect that the antibody will exert the same pattern of therapeutic activity in AD patients.

## Conclusion

We have identified a novel therapeutic target on tau protein that can be utilised for the treatment of AD. On the basis of our study findings, we can draw the following conclusions. (1) The HXPGGG amino acid sequence represents minimal epitope, structural determinant on mis-disordered protein tau recognised by DC8E8. (2) The minimal DC8E8 epitope on mis-disordered human tau is present four times as four separate structural determinants, epitopes, each encompassing six amino acids. (3) The primary structure of epitope 1 is ^268^HQPGGG^273^ (located within MTBR1), that of epitope 2 is ^299^HVPGGG^304^ (located within MTBR2), that of epitope 3 is ^330^HKPGGG^335^ (located within MTBR3) and that of epitope 4 is ^362^HVPGGG^367^ (located within MTBR4) (Figure 5C). (4) DC8E8 recognises mis-disordered tau with far higher affinity than physiological tau. Mis-disordered tau displays a DC8E8 high-affinity epitope, and physiological tau displays a DC8E8 low-affinity epitope. (5) Binding of DC8E8 to a high-affinity epitope inhibits pathological tau–tau interaction and thus delineates a key regulatory domain for pathological tau assembly. Binding of DC8E8 to a low-affinity epitope on physiological tau has no impact on its physiological function (promotion of microtubule assembly). The discriminatory potential of DC8E8 in targeting of diseased and healthy tau underlines its use as a safe immunotherapeutic agent. (6) DC8E8 high-affinity epitope represents the most sensitive and vulnerable target for AD immunotherapy identified to date. (7) Because formation of DC8E8 high-affinity epitope is an essential event for pathological transition of tau and is present on every diseased tau molecule, we suggest naming this druggable structure the ‘Achilles heel of diseased tau’.

## Abbreviations

AD: Alzheimer’s disease; AU: Asymmetric unit; CDR: Complementarity determining region; ELISA: Enzyme-linked immunosorbent assay; Fab: Fragment antigen-binding; HRP: Horse radish peroxidase; mAb: Monoclonal antibody; MTBR: Microtubule-binding repeat; NCS: Noncrystallographic symmetry; NFT: Neurofibrillary tangle; PBS: Phosphate-buffered saline; PDB: Protein Data Bank; PVDF: Polyvinylidene fluoride; RU: Response unit; SPR: Surface plasmon resonance.

## Competing interests

EK, NZ, BK, RS and MN are employees of Axon Neuroscience SE and do not own any shares of the company. The authors have no other competing interests to declare.

## Authors’ contributions

EK created the DC8E8 antibody and performed most of the immunological analyses, including interpretation of data. NZ performed animal efficacy studies and histological analyses, including interpretation of data. BK performed tau–tau interaction studies, including interpretation of data. RS performed crystallographic and surface plasmon resonance analyses, including interpretation of data. MN conceived of and designed the study and drafted the manuscript. All authors read and approved the final manuscript.

## Supplementary Material

Additional file 1: Table S1 Details of diffraction data collection and processing statistics of DC8E8 Fab apo-form. Click here for file

Additional file 2: Table S2 Refinement statistics of the structure of DC8E8 Fab apo-form. Click here for file
