# Supplementary material for: Identification of structural determinants on tau protein essential for its pathological function: novel therapeutic target for tau immunotherapy in Alzheimer’s disease
Source: Alzheimers Res Ther. 2014 Aug 1;6(4):45. doi: 10.1186/alzrt277 (PMC4255369; doi:10.1186/alzrt277)
Supplement: Additional file 1: Table S1 — Details of diffraction data collection and processing statistics of DC8E8 Fab apo-form. [file alzrt277-S1.doc]

Supplementary Table 1

Details of diffraction data collection and processing statistics of DC8E8 Fab apo-form

| **Diffraction source** | **X06DA** |
| --- | --- |
| **Wavelength (Å)** | 1.0000 |
| **Temperature (K)** | 100 |
| **Detector** | MarCCD (225 mm) |
| **Crystal-detector distance (mm)** | 320 |
| **Rotation range per image (°)** | 0.5 |
| **Total rotation range (°)** | 95 |
| **Exposure time per image (s)** | 2.5 |
| **Space group** | P 21 |
| **a, b, c (Å)** | 41.11, 110.89, 95.76 |
| **β (°)** | 90.48 |
| **Mosaicity (°)** | 0.524 |
| **Resolution range (Å)** | 39.54-3.00 (3.07-3.00) |
| **Total No. of reflections** | 34215 (2452) |
| **No. of unique reflections** | 14374 (1076) |
| **Completeness (%)** | 83.3 (86.1) |
| **Multiplicity** | 2.4 (2.3) |
| **<*I*/σ(*I*)>** | 7.89 (2.06) |
| **R_r.i.m._(%)*** | 15.7 (57.8) |
| **CC_1/2_ (%)**** | 98.5 (76.8) |
| **Overall B factor from Wilson plot (Å^2^)** | 55.28 |
| **Matthews coefficient (Å^3^/Dalton)** | 2.27 |
| **Solvent content (%)** | 45.9 |
| **Monomers in asymmetric unit** | 2 |

* R_r.i.m_. (R_meas_) is defined as$\sum_{hkl} \left\{N(hkl)/\left.\left[N(hkl)-1\right]\right\}\right.^{1/2}\sum_{i} \left|I_{i}\right.(hkl)-\left.\left\langle\left.I(hkl)\right\rangle\right.\right|/\sum_{hkl} \sum_{i} I_{i}(hkl)$where I_i_(hkl) is the intensity of i^th^ observation of reflection hkl, $\left\langle\left.I(hkl)\right\rangle\right.$is the average of N observations of reflection hkl.

**percentage of correlation between intensities from random half-datasets
