# Supplementary material for: Identification of structural determinants on tau protein essential for its pathological function: novel therapeutic target for tau immunotherapy in Alzheimer’s disease
Source: Alzheimers Res Ther. 2014 Aug 1;6(4):45. doi: 10.1186/alzrt277 (PMC4255369; doi:10.1186/alzrt277)
Supplement: Additional file 2: Table S2 — Refinement statistics of the structure of DC8E8 Fab apo-form. [file alzrt277-S2.doc]

Supplementary Table 2

Refinement statistics of the structure of DC8E8 Fab apo–form

| **Resolution (Å)** | **3.0** |
| --- | --- |
| R_work_(%) | 23.0 |
| R_free_(%) | 27.9 |
| **Number of non-hydrogen atoms** | |
| Fab heavy chains | 3309 |
| Fab light chains | 3371 |
| Waters | 8 |
|  |  |
| **Average B factors (Å^2^)** | |
| Fab heavy chains | 57.4 |
| Fab light chains | 65.5 |
| Waters | 30.3 |
|  |  |
| **RMSD from ideal geometry** | |
| Bond lengths (Å) | 0.0090 |
| Bond angles(°) | 1.3724 |
| **Ramachandran plot analyzed with RAMPAGE (%)** | |
| Favored | 96.5 |
| Allowed | 3.3 |
| Disallowed | 0.1 |
